# Relationship between plasma circulating cell-free DNA concentration and treatment outcomes including prognosis in patients with advanced non-small cell lung cancer

**DOI:** 10.1186/s12890-023-02586-2

**Published:** 2023-09-14

**Authors:** Wei-Wei Peng, Ying Liu, Huan-Huan Sha, Shao-Di Wen, Ying Fang, Guo-Ren Zhou

**Affiliations:** Jiangsu Cancer Hospital, Jiangsu Institute of Cancer Research, The Affiliated Cancer Hospital of Nanjing Medical University, Baiziting 42, Nanjing, 210009 Jiangsu China

**Keywords:** Non-small cell lung cancer (NSCLC), Plasma cell-free DNA (cfDNA), Prognosis biomarkers

## Abstract

**Background:**

Some research found that elevated plasma cell-free DNA (cfDNA) concentrations and poor prognosis are associated in non-small cell lung cancer (NSCLC). However, more studies need to be carried out to verify this conclusion. Therefore, this study investigated the relationship between cfDNA concentration and treatment outcomes including prognosis in patients with advanced NSCLC.

**Methods:**

We retrospectively collected medical records and cfDNA data from 160 patients with advanced NSCLC. Progression-free survival (PFS) were calculated using the Kaplan-Meier method and were compared between groups using the log rank test. Cox regression analysis was used for estimating the independent predictors of PFS. And we used logistic regression to evaluate the relationship between baseline biomarkers and efficacy. In our study, BT1 cfDNA, BT2 cfDNA, and BT3 cfDNA were defined as cfDNA concentration before the first treatment (baseline cfDNA concentration), cfDNA concentration before the second treatment, and cfDNA concentration before the third treatment, respectively.

**Results:**

Patients with low cfDNA (BT1 cfDNA < 15 (ng/mL)) were reported a significantly prolonged median progression-free survival (mPFS) compared with patients with patients with high cfDNA (BT1 cfDNA ≥ 15(ng/mL)) (mPFS: 14.6 vs. 8.3 months, *P* = 0.002), as well as patients with neutrophil/lymphocyte ratio (NLR)<2.98 (mPFS: 13.1 vs. 7.9 months, *P* = 0.023). In addition, Cox proportional hazards regression analysis identified independent indicators associated with PFS including BT1 cfDNA ≥ 15 (ng/mL), NLR ≥ 2.98 and extrapulmonary metastasis. The best cut-off value for BT3 cfDNA for predicting disease progression is 41.46 (ng/mL) (Area Under the Curve (AUC): 0.652, *95%CI*: 0.516–0.788), achieving 90.7% sensitivity and 37.5% specificity for the prediction of disease progression. BT3 cfDNA (OR = 6.08, *95% CI*: 1.94–19.57, *P* = 0.002) was an independent factor for disease progression in patients with advanced NSCLC.

**Conclusions:**

BT1 cfDNA may be a biomarker to assess the prognosis of advanced NSCLC. Patients with advanced NSCLC with lower cfDNA and NLR before treatment had a better prognosis. Increased BT3 cfDNA concentration was an independent factor of disease progression in advanced NSCLC patients. These findings may assist in identifying high-risk patients and guiding treatment strategies.

**Supplementary Information:**

The online version contains supplementary material available at 10.1186/s12890-023-02586-2.

## Introduction

Lung cancer was the second most commonly diagnosed cancer and the leading cause of cancer-related death worldwide in 2020[1]. Non-small cell lung cancer (NSCLC) accounts for 80–85% of lung cancer, with a very high incidence and mortality. Based on histology, staging, genomics, and the patient’s condition, the current treatment options for NSCLC include surgery, radiotherapy, chemotherapy, immunotherapy, and molecular targeted therapy[2]. However, most patients are in the advanced stage when diagnosed, and palliative care is the only option for these patients. Therefore, the prognosis of patients with advanced NSCLC is often poor. Upon these, it is of great importance to actively explore novel prognostic biomarkers for advanced NSCLC patients to guide treatment decision-making to improve the survival of patients.

Over the past decade, traditional biomarkers have shown low sensitivity and specificity in predicting the diagnosis, efficacy and prognosis of NSCLC patients[3]. It is urgent to seek a non-invasive, minimally invasive, highly reproducible marker that can predict prognosis and dynamically monitor efficacy in NSCLC. Cell-free DNA (cfDNA) consists of short (≈ 160 (nt)) double-stranded DNA segments that are continuously cleared from bloodstrea (cfDNA has a half-life of 5-150 min)[4]. Thus, cfDNA is a “snapshot” of dying cells throughout the body and can be used to detect a wide and diverse range of biomarkers for a variety of diseases[5–7]. In particular, cfDNA has gained traction in cancer diagnosis over the past five year[8–10]. cfDNA is found mainly in blood, but also in other body fluids such as urine, pleural fluid, and cerebrospinal fluid[11]. The mechanisms of apoptosis, tumor cell necrosis, active release and lysis of circulating tumor cells have been proposed. Plasma cfDNA levels in cancer patients are often higher than those in healthy people. In addition, studies of NSCLC patients have found that higher cfDNA levels are significantly associated with poor prognosis in patients of NSCLC[12–14]. However, the predictive effect of cfDNA on prognosis and efficacy remains controversial and needs to be explored in more studies[15].

In this study, we aimed to clarify the relationship between cfDNA concentration and treatment outcomes including prognosis in patients with advanced NSCLC.

## Materials and methods

### Patient population

In this retrospective study, 160 stage IIIB-IV NSCLC patients treated at Jiangsu Cancer Hospital between January 2018 and January 2021 were included for analysis, and plasma cfDNA of the patients were analyzed.

Patients were treated according to the Declaration of Helsinki’s ethical principles for medical research involving human subjects. Patients were eligible if they were ≥ 18 years, histologically or cytologically confirmed NSCLC, stage IIIB-IV (according to version 8 of the AJCC), ECOG-PS 0–2 and not received clinical treatment or the interval of treatment for more than half a year, with baseline plasma cfDNA concentration, peripheral blood white blood cells, neutrophils, and lymphocyte counts available before treatment.

Patients were excluded if they were in acute infection, combined with severe impairment of organ function and diseases of the blood and autoimmune system, non-primary or combined with other malignancies, had incomplete baseline data, had less than two cycles of therapy, and did not have pre- and post-treatment imaging.

### Sample collection and cfDNA extraction

Clinical data and routine blood tests which included blood cells, neutrophils and lymphocyte count were collected from patient charts and medical records at Jiangsu Cancer Hospital. neutrophil/lymphocyte ratio (NLR) was neutrophil/lymphocyte ratio.

Fasting venous blood was drawn from patients respectively one day before the first cycle of treatment, the second cycle of treatment and the third cycle of treatment. Each time approximately 2–4 (mL) of peripheral venous blood was collected using EDTA-K2 anticoagulation tubes. The peripheral venous blood was centrifuged at 3000 (g) for 10 min within 6 h. The plasma layer of about 1.5 (mL) was diluted with PBS buffer and the supernatant was stored by centrifugation at 1300 (g). The cfDNA was extracted from plasma using QuantiDNA Direct cfDNA Test (DiaCarta) according to manufacturer’s protocol. BT1 cfDNA, BT2 cfDNA, and BT3 cfDNA were defined as cfDNA concentration before the first treatment (baseline cfDNA concentration), cfDNA concentration before the second treatment, and cfDNA concentration before the third treatment, including immunotherapy, chemotherapy or targeted therapy, respectively. BT2-BT1 cfDNA、BT3-BT1 cfDNA was defined as the difference in cfDNA concentration before the second treatment and before the first treatment, and the difference in cfDNA concentration before the third treatment and before the first treatment.

Radiological evaluation of treatment efficacy by computed tomography (CT) scan was performed during treatment in our hospital, thereafter until disease progression and responses were evaluated by the response evaluation criteria in solid tumors (RECIST) version 1.1.

### Statistical analysis

The Kolmogorov-Smirnov test was used for all continuous variables to test the hypothesis of normal distribution. We assessed correlations between BT1 cfDNA and continuous variables by using Spearman correlation analysis. Optimal cutoff values for both BT1 cfDNA and NLR were calculated by the survminer package surv cutpoint function. The Kaplan-Meier method was used to explore the prognostic effect of BT1 cfDNA and NLR on progression-free survival (PFS), and the log-rank test to count the difference in survival probability between groups. Cox Proportional Hazard Model were used to investigate independent prognostic factors for PFS (all preconditioning parameters with *P* < 0.05 found in univariate analyses were included as covariates in multivariate analyses). Multiple logistic regression models estimated the relationship between different efficacy groups (including progressive disease (PD), partial response (PR), stable disease (SD)) and BT1 cfDNA and NLR levels, with all statistics were performed in R. *P* < 0.05 was considered statistically significant. The factors affecting disease progression were analyzed by generalized linear regression model.

We calculated the sample size of the multivariable Cox regression model for PFS using the previously reported method. Given the widely accepted rule of thumb of 20 events per variable and given there were 3 variables in the final Cox model, the total number of events expected was 60. Taking into account an approximate 64% 1-year event rate and a 20% lost-to-review rate among the participants, we required a total sample size of at least 117 patients.

## Results

### Baseline characteristics

Patient characteristics are summarized in Table 1. A total of 160 patients were enrolled, and the median age was 61.58 years old. The screening process and results are shown in Supplementary Figure S1.The pathological type of most patients was adenocarcinoma (78.1%). 57 patients received previous chemotherapy alone (35.6%). 63 patients (39.4%) received chemotherapy and Bevacizumab, 33 patients (20.6%) received immunotherapy, and 7 patients (4.4%) received oral targeted agents.

**Table 1:** Baseline characteristics of patients

Characteristic	Total(n = 160)	%	
Sex			
Male	114	71.3	
Female	46	28.7	
Age			
<65	87	54.4	
≥ 65	73	45.6	
Smoking history			
Current/former	40	25.0	
Never	120	75.0	
ECOG PS			
0	15	9.4	
1	135	84.4	
2	10	6.2	
Histology			
Adenocarcinoma	125	78.1	
Squamous cell carcinoma	31	19.4	
Other	4	2.5	
Degree of differentiation			
Low	98	61.3	
Low-medium	29	18.1	
Medium-high	28	17.5	
High	5	3.1	
Clinical stage at diagnosis			
IIIB-C	21	13.1	
IV	139	86.9	
Metastatic sites			
Intrapulmonary	82	51.3	
Extrapulmonary	78	48.7	
Tumor size			
<5 cm	99	61.9	
≥ 5 cm	46		28.8
Unable to measure	15	9.3	
Treatment strategies			
Mono chemotherapy	57	35.6	
Chemotherapy and Bevacizumab	63	39.4	
Immunotherapy	33	20.6	
Targeted therapy	7	4.4	

Information including absolute values of white blood cells (WBC), neutrophils (NEU), lymphocytes (LYM), NLR, BT1, BT2, and BT3 cfDNA concentrations in routine blood tests is shown in Table 2, mPFS of patients: 10.37 months (*95% CI*: 8.37–13.2), median follow-up time:17.5 months (*95% CI*: 13.6–25.4).

**Table 2:** Baseline characteristics of patients

Characteristic	M*(P*_*25*_, *P*_*75*_*)*
WBC	6.74(5.58, 8.35)
NEU	4.38(3.61, 5.96)
LYM	1.59(1.29, 1.99)
NLR	2.85(2.01, 4.07)
BT1 cfDNA	20.62(13.15, 33.17)
BT2 cfDNA	19.65(11.79, 30.06)
BT3 cfDNA	19.80(11.76, 27.62)

### Correlation between BT1 cfDNA concentration and clinical characteristics

**Fig. 1:**
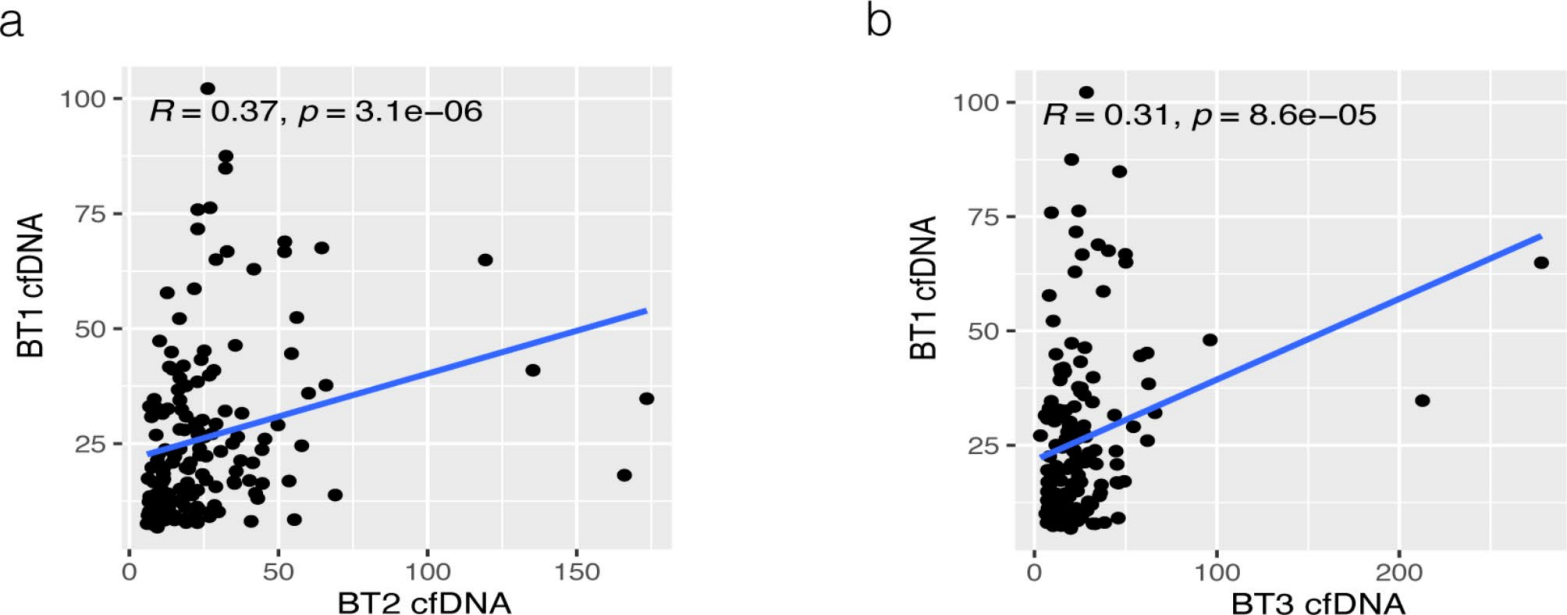
Correlation of BT1 cfDNA and BT2 cfDNA、BT3 cfDNA (**a**) Spearman analysis of BT1 and BT2 (**b**) Spearman analysis of BT1 and BT3

**Fig. 2:**
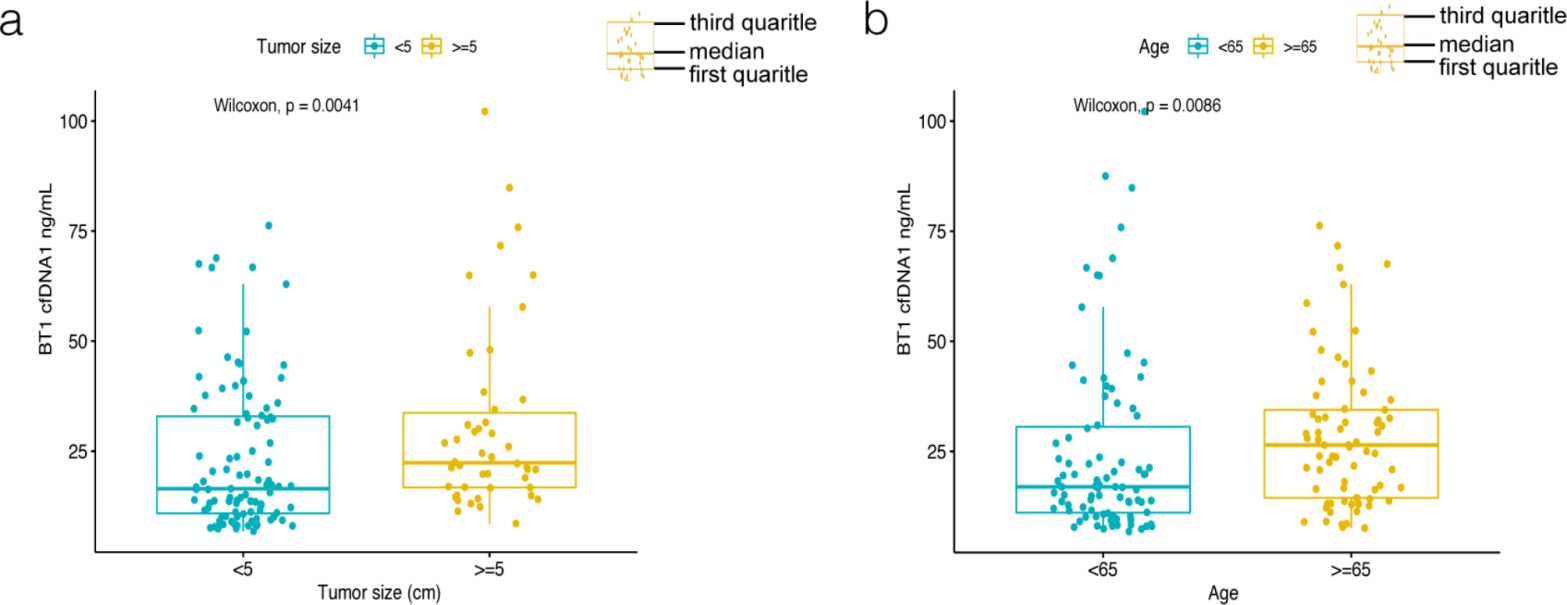
BT1 cfDNA concentration in different tumor size (**a**) and age groups (**b**) cfDNA, circulating free DNA.

In plasma samples respectively collected from 160 NSCLC patients, cfDNA concentrations ranged from 3.24 to 277.90 (ng/mL). Spearman analysis showed that BT2 and BT3 were significantly correlated with BT1. (Fig. 1a, b). When comparing the relationship between BT1 cfDNA and clinicopathological features, we found higher cfDNA concentrations in the subgroup with tumor size ≥ 5 (cm) (median 22.37 vs. 16.48 (ng/mL), *P* = 0.004) and age ≥ 65 (median 26.45 vs. 16.97 (ng/mL), *P* = 0.009). (Fig. 2, Supplementary Table S1)

### Prognostic of BT1 cfDNA and NLR

BT1 cfDNA and NLR analyses were performed in 160 patients at baseline. The optimal cutoff values for BT1 cfDNA and NLR is 15 (ng/mL) and 2.98.(Supplementary Figure S2a, c). The mPFS of patients with high BT1 cfDNA (BT1 cfDNA ≥ 15(ng/mL)) was 8.3 months and 14.6 months in patients with low BT1 cfDNA (BT1 cfDNA < 15(ng/mL)) (Log-rank *P* = 0.002, HR = 1.97 *95Cl*: 1.29–3.01). The mPFS of patients with high NLR at baseline (NLR ≥ 2.98) was 7.9 months and 13.1 months in patients with low NLR (NLR<2.98) (Log-rank *P* = 0.023, HR = 1.57 *95Cl*: 1.06–2.32). (Supplementary Figure S2b, d)

### Analyses of prognostic biomarkers in advanced NSCLC

**Fig. 3:**
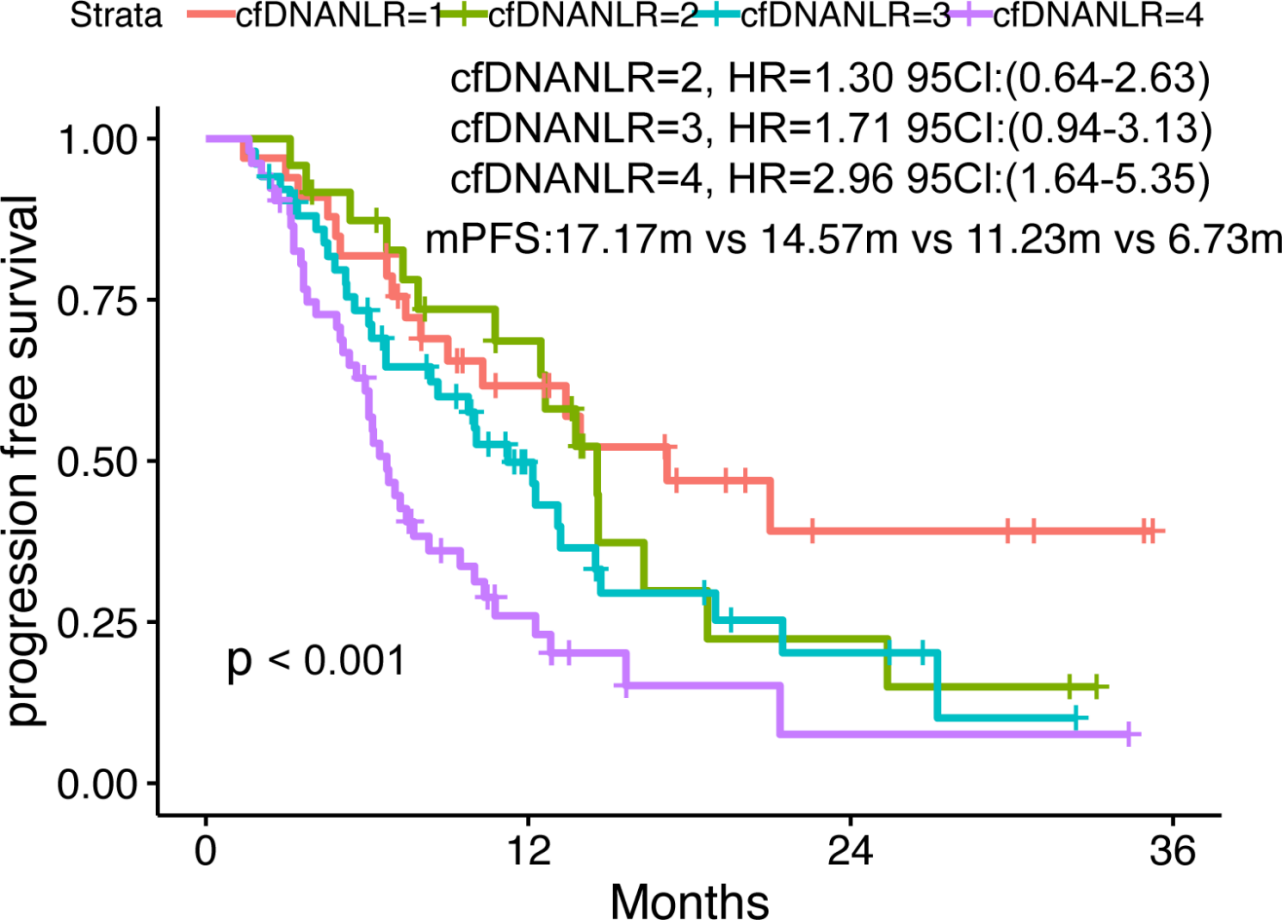
Kaplan-Meier analysis of the BT1 cfDNA/NLR subgroup. 1: “Low grade” ((BT1 cfDNA < 15ng/mL and NLR < 2.98); 2: “Low-intermediate” (BT1 cfDNA < 15ng/mL and NLR ≥ 2.98); 3: " High Intermediate” (BT1 cfDNA ≥ 15ng/mL and NLR < 2.98); 4: “High Grade” (BT1 cfDNA ≥ 15ng/mL and NLR ≥ 2.98)

In the univariate Cox regression model analysis, we found that the extrapulmonary metastases, tumor size, BT1 cfDNA and NLR were associated with PFS (*P*<0.05). Then, Multivariable analysis identified three significant factors associated with independent PFS: cfDNA ≥ 15 (ng/mL), (HR: 1.98, 95%*CI*: 1.26–3.11, *P* = 0.003), NLR ≥ 2.98 (HR:1.86, 95% *CI*: 1.22–2.84, *P* = 0.004) and extrapulmonary metastases (HR: 1.68, 95%*CI*: 1.10–2.55, *P* = 0.016) (Table 3). Upon this, we combined BT1 cfDNA and NLR into the following four risk subgroups: 1: “Low grade” (BT1 cfDNA < 15 (ng/mL) and NLR < 2.98); 2: “Low-intermediate” (BT1 cfDNA < 15 (ng/mL) and NLR ≥ 2.98); 3: " High Intermediate” (BT1 cfDNA ≥ 15 (ng/mL) and NLR < 2.98); 4: “High Grade” (BT1 cfDNA ≥ 15 (ng/mL) and NLR ≥ 2.98). Supplementary Figure S3 contains the results of univariate Log-rank tests and mPFS for each variable group. Analysis of the four risk subgroups revealed that patients in the high-risk subgroup had a significantly worse prognosis compared to those in the low-risk subgroup, with nearly threefold the risk of progression compared to the low-risk subgroup (HR = 2.96, *95Cl*: 1.64–5.35, *P* < 0.001) (Supplementary Table S2, Fig. 3). The mPFS of the four subgroups were 17.17 m, 14.57 m, 11.23 m, and 6.73 m, respectively.

**Table 3:** The relationship between demographic and clinical variables and survival

Variables	Univariate			Multivariate		
	HR	*95%CI*	*P* value	HR	*95%CI*	*P* value
Gender (Female)	0.94	(0.61–1.45)	0.770			
Age (≥ 65)	0.86	(0.58–1.28)	0.461			
Smoking history (Yes)	0.99	(0.64–1.54)	0.974			
ECOG PS (1–2)	1.07	(0.54–2.13)	0.837			
Primary site (Right)	0.73	(0.49–1.07)	0.105			
Histology(Squamous cell carcinoma and others)	1.55	(0.99–2.42)	0.056			
Degree of differentiation (High)	1.19	(0.76–1.85)	0.456			
Stage (IV)	0.70	(0.40–1.24)	0.225			
Extrapulmonary metastasis (Yes)	1.50	(1.02–2.22)	0.042	1.68	(1.10–2.55)	0.016
Tumor size (≥ 5(cm))	1.57	(1.02–2.40)	0.041	1.43	(0.93–2.21)	0.100
BT1 cfDNA (≥ 15(ng/mL))	1.97	(1.29–3.01)	0.002	1.98	(1.26–3.11)	0.003
NLR (≥ 2.98)	1.57	(1.06–2.32)	0.024	1.86	(1.22–2.84)	0.004

### Relationship between dynamics of cfDNA levels and treatment efficacy

**Fig. 4:**
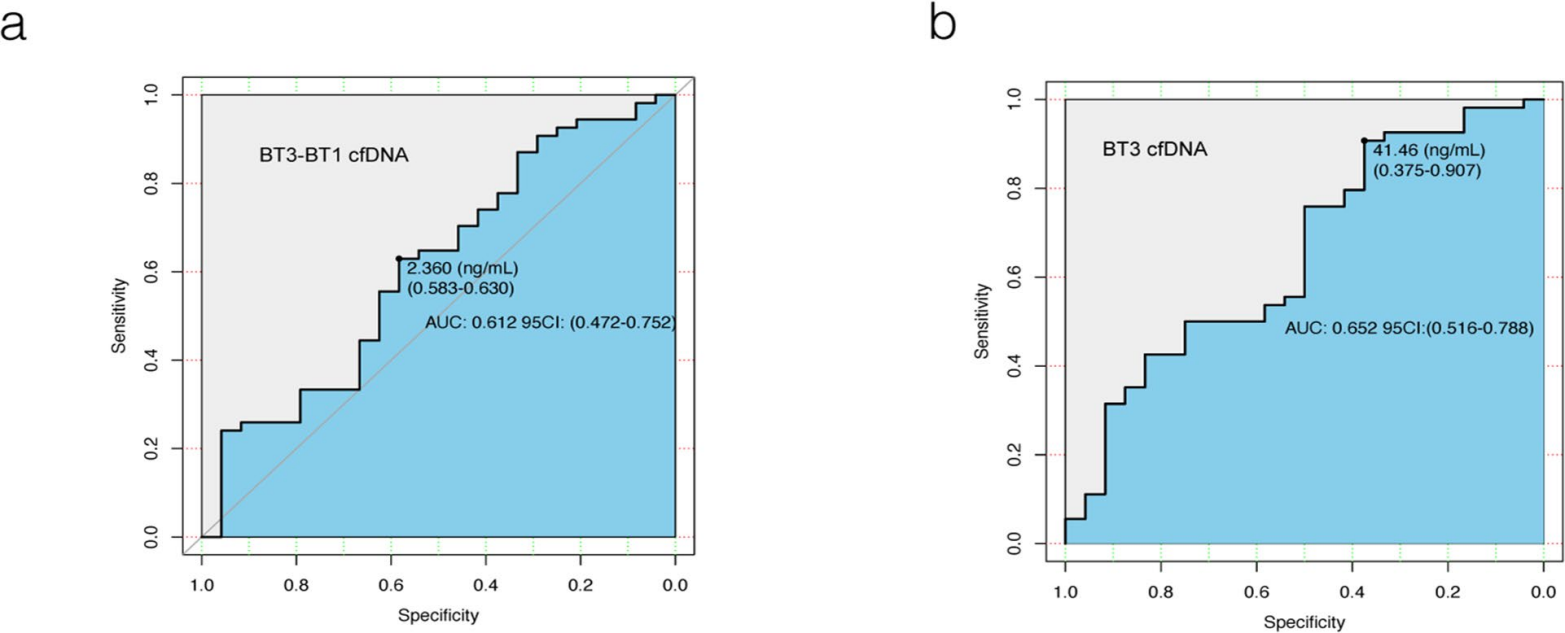
ROC diagnostic efficacy of cfDNA (BT3, BT3-BT1) concentration to predict disease progression

The disease control rate (DCR) of the 160 patients enrolled was 85% (54 PR and 82 SD), and the remaining 24 patients were PD. Median cfDNA concentrations (BT1, BT2, BT2-BT1, BT3-BT1) did not differ between the three groups (PD, PR and SD). In addition, there was simply no statistical difference in BT3 among the three groups (*P* = 0.048), though patients in the PD group had higher BT3 cfDNA concentrations than those in the PR and SD groups (Supplementary Table S3, Supplementary Figure S4). When patients were divided into disease control (PR + SD) and PD groups,BT3 cfDNA concentrations in the (PR + SD) (median 19.10 (ng/mL)) and PD (median 24.96 (ng/mL)) groups were found to be statistically different (*P* = 0.020) (Supplementary Table S4 and S5). The diagnostic efficacy of BT3 cfDNA concentration and BT3-BT1 cfDNA concentration in predicting disease progression was shown in Fig. 4. BT3 cfDNA concentration was more effective in identifying PD patients with 90.7% sensitivity and 37.5% specificity(AUC: 0.652, *95%CI*: 0.516–0.788, cut-off value 41.46 (ng/mL)). The results demonstrated that BT3 cfDNA concentration could be predictive of disease progression in patients with advanced NSCLC.

### Factors associated with disease progression in patients with advanced NSCLC

We used logistic regression models to explore the factors associated with disease progression in patients with advanced NSCLC. We found that BT3 cfDNA and BT3-BT1 cfDNA concentrations were associated with disease progression (P < 0.05).We further found that BT3 cfDNA (OR: 6.08, *95% CI*: 1.94–19.57, *P* = 0.002) was an independent factor affecting disease progression in patients with advanced NSCLC (Table 4).

**Table 4:** Association between cfDNA levels and clinicopathological characteristics and survial

Variables	Univariate			Multivariate		
	OR	95%CI	*P* value	OR	95%CI	*P* value
Gender (Female)	0.61	(0.19–1.64)	0.356			
Age (≥ 65)	1.23	(0.51–2.96)	0.641			
Smoking history (Yes)	2.03	(0.79–5.03)	0.130			
ECOG PS (1–2)	0.68	(0.19–3.15)	0.571			
Primary site (Right)	0.84	(0.35–2.01)	0.690			
Histology (Squamous cell carcinoma and others)	0.93	(0.29–2.54)	0.894			
Degree of differentiation (High)	0.74	(0.20–2.14)	0.602			
Stage (IV)	0.71	(0.23–2.67)	0.579			
Extrapulmonary metastasis (Yes)	0.71	(0.29–1.71)	0.453			
Tumor size (≥ 5(cm))	1.84	(0.72–4.57)	0.191			
BT3 cfDNA (≥ 41.46(ng/mL))	3.06	(2.61–21.71)	< 0.0001	6.08	(1.94–19.57)	0.002
BT3-BT1 cfDNA (≥ 2.36(ng/mL))	2.62	(1.09–6.52)	0.032	1.54	(0.55–4.21)	0.398

## Discussion

In this retrospective study, we found that BT1 cfDNA levels was an independent prognostic indicator for advanced NSCLC patients. In addition, we revealed that patients with advanced NSCLC with increased levels of both BT1 cfDNA and NLR had a worse prognosis. We also revealed that BT3 cfDNA could identify patients with advanced NSCLC who may have disease progression and could be used as a simple and convenient monitoring indicator.

The search for mechanisms and prognostic markers of tumors has been ongoing[16–19]. The predictive role of cfDNA in prognosis has been demonstrated in many studies in recent years. These studies have important clinical implications that serum samples were often obtained as part of routine blood tests, potentially eliminating the need for additional samples for cfDNA analysis. This study demonstrated that baseline cfDNA levels could be used as an independent prognostic indicator for advanced non-small cell lung cancer, which is consistent with some previous findings that elevated plasma cfDNA concentrations and poor prognosis are associated [20–24]. Hyun et al[25] found that high cfDNA concentrations may be an independent predictor of PFS and OS in NSCLC. A recent study revealed that the lack of increase in ctDNA levels was linked to a considerably longer PFS (median: 0.7 months versus 12.0 months)[26]. Our present study focused on the inclusion of stage IIIB-IV NSCLC patients, and the optimal stage value of BT1 cfDNA was calculated to be 15 (ng/mL), and patients of high BT1 cfDNA group had better PFS, suggesting that dynamic monitoring of cfDNA may be beneficial in predicting NSCLC outcomes. cfDNA is mainly present in serum and plasma together with histones as nucleosomes and shows high potential for diagnostic, prognostic and therapeutic monitoring [20]. In a human NSCLC xenograft mouse model, cfDNA showed a significant transient increase immediately after chemotherapy or surgery, followed by a rapid decrease [27]. The level of cfDNA in peripheral blood provided a real-time snapshot of tumor cell death as well as an indirect measure of overall tumor load, making it a reliable surrogate indicator of tumor treatment response. Compared to expensive imaging tools, cfDNA analysis is cost-effective and continuous monitoring of cfDNA may have a potential role in monitoring tumor efficacy.

The role of cfDNA and NLR in the prognosis of advanced NSCLC has been demonstrated separately [28–30].However, in this study, we identified a high-risk subgroup with both high cfDNA and NLR by combining the two biomarkers, and found that they had the highest risk of disease progression. The complementary effects of these two biomarkers suggest the potential of using cfDNA and NLR in combination to predict prognosis in NSCLC patients in clinical practice. This provides insight into the significance of combining multiple metrics for prediction, as the application of a single marker for prediction may not meet clinical needs. We found several independent prognostic factors such as cfDNA, NLR and tumor size in advanced NSCLC patients, which is consistent with previous research [31–33]. One study [34] demonstrated that monitoring cfDNA changes and pretreatment PA levels in advanced NSCLC patients receiving treatment is an accurate predictor of tumor response and PFS. Therefore, all this evidence reveals the importance for every clinician to comprehensively identify independent prognostic factors and individually evaluate the prognosis of patients.

The association between high cfDNA concentrations and poor prognosis may be related to tumor load or comorbidities. Our study is limited only to the quantification of cfDNA and should also be analyzed for tumor-specific genomic alterations in cfDNA such as EGFR[35] and KRAS [36] for more precise efficacy prediction and personalized treatment selection. In addition, advanced lung adenocarcinoma might have a higher level of heterogeneity[37], but we did not refine the patient’s treatment plan, which may have affected the accuracy of the results. We did not follow up further on patient survival and lacked Overall survival (OS) data, which should have been further analyzed. It is also important to mention here that since this retrospective study only involved a limited number of NSCLC patients, the conclusions still need to be validated by more external, multicenter and large-scale prospective studies.

In conclusion, our data suggest that high baseline cfDNA concentrations in plasma are associated with poor prognosis in advanced NSCLC. Baseline cfDNA concentrations perform poorly in assessing NSCLC efficacy and do not provide an accurate prediction of efficacy. However, increased BT3 cfDNA in PD patients may be used as an adjunct to imaging to assess efficacy. This may help in the adjustment of treatment regimens in some patients when progression is not yet clear on CT imaging.

### Electronic supplementary material

Below is the link to the electronic supplementary material.

Supplementary Material 1

Supplementary Material 2

Supplementary Material 3

Supplementary Material 4

Supplementary Material 5

Supplementary Material 6

Supplementary Material 7

Supplementary Material 8

Supplementary Material 9

## Data Availability

The datasets generated during and/or analyzed during the current study are available from the corresponding author on reasonable request.
