# Supplementary material for: Relationship between plasma circulating cell-free DNA concentration and treatment outcomes including prognosis in patients with advanced non-small cell lung cancer
Source: BMC Pulm Med. 2023 Sep 14;23:348. doi: 10.1186/s12890-023-02586-2 (PMC10503004; doi:10.1186/s12890-023-02586-2)
Supplement: Supplementary file 1 — Supplementary Material 1 [file 12890_2023_2586_MOESM1_ESM.docx]

Supplementary Fig S1. Flowchart of the study.

Supplementary Fig S2. BT1 cfDNA concentration and the relationship between NLR and PFS. (a) BT1 cfDNA optimal cut-off value and high and low grouping distribution; (b) Kaplan-Meier curve using the optimal cut-off value of BT1 cfDNA determined by a; (c) NLR optimal cut-off value and distribution of high and low groups; (d) Kaplan-Meier curves using the NLR optimal cutoff values determined by c.

Supplementary Fig S3. KM graphs and mPFS of each variable group.

Supplementary Fig S4 cfDNA levels of BT1, BT2 and BT3 among PD, PR and SD groups.
