# Supplementary material for: Relationship between plasma circulating cell-free DNA concentration and treatment outcomes including prognosis in patients with advanced non-small cell lung cancer
Source: BMC Pulm Med. 2023 Sep 14;23:348. doi: 10.1186/s12890-023-02586-2 (PMC10503004; doi:10.1186/s12890-023-02586-2)
Supplement: Supplementary file 2 — Supplementary Material 2 [file 12890_2023_2586_MOESM2_ESM.docx]

**Supplementary Appendix**

Supplementary Table S1 Relationship between BT1 cfDNA concentration and clinicopathological characteristics of NSCLC patients

| Characteristic | Total（n=160） | BT1 cfDNA（ng/mL） | *p* value |
| --- | --- | --- | --- |
| Gender |  |  |  |
| Male | 114 | 19.78（13.17, 31.58） | 0.604 |
| Female | 46 | 21.52（13.10, 36.52） |  |
| Age |  |  |  |
| ＜65 | 87 | 16.97（11.09, 30.61） | 0.009 |
| ≥65 | 73 | 26.45（14.44, 34.44） |  |
| ECOG PS |  |  |  |
| 0 | 15 | 17.11（13.85，22.04） | 0.168 |
| 1 | 135 | 20.91（12.90，34.53） |  |
| 2 | 10 | 31.00（14.41，49.72） |  |
| Smoking history |  |  |  |
| Yes | 40 | 22.35（16.91，32.82） | 0.104 |
| No | 120 | 19.82（11.95，33.70） |  |
| Histology |  |  |  |
| Adenocarcinoma | 125 | 20.43（12.36，33.08） | 0.709 |
| Squamous cell carcinoma | 31 | 21.30（14.13，36.53） |  |
| Other | 4 | 20.10（15.88，25.50） |  |
| Stage |  |  |  |
| IIIB-C | 21 | 17.11（13.72，26.87） | 0.986 |
| IV | 139 | 20.86（13.03，33.27） |  |
| Metastatic sites |  |  |  |
| Intrapulmonary | 82 | 20.10（13.53，31.98） | 0.985 |
| Extrapulmonary | 78 | 20.89（12.13，37.15） |  |
| NLR |  |  |  |
| ＜2.98 | 84 | 23.47（11.96，31.09） | 0.131 |
| ≥2.98 | 76 | 29.47（13.91，39.86） |  |
| Tumor size |  |  |  |
| ＜5cm | 99 | 16.48（10.87，32.91） | 0.004 |
| ≥5cm | 46 | 22.37（16.74，33.71） |  |
| Unable to measure | 15 | 27.98（25.19，35.71） |  |

Supplementary Table S2 COX analysis of cfDNA/NLR combination

| Subgroup | BT1 cfDNA | NLR | HR | 95%*CI* | *P Value* |
| --- | --- | --- | --- | --- | --- |
| 1 | ＜15ng/mL | ＜2.98 | 1 | Ref | Ref |
| 2 |  | ≥2.98 | 1.56 | （0.58～2.82） | 0.55 |
| 3 | ≥15ng/mL | ＜2.98 | 1.13 | （0.79～3.07） | 0.20 |
| 4 |  | ≥2.98 | 3.33 | （1.79～6.21） | <0.001 |

HR is a risk ratio adjusted for sex, age, smoking history, primary site, pathological type, degree of differentiation, tumor size, ECOG PS, stage, and metastatic status.

1: "Low grade" (BT1 cfDNA <15ng/mL and NLR <2.98); 2: "Low-intermediate" (BT1 cfDNA <15ng/mL and NLR ≥2.98); 3: " High Intermediate" (BT1 cfDNA ≥15ng/mL and NLR <2.98); 4: "High Grade" (BT1 cfDNA ≥15ng/mL and NLR ≥2.98)

Supplementary Table S3 RECIST criteria to assess the relationship between efficacy and cfDNA concentration

| cfDNA （ng/mL） | PD | PR | SD | *P* Value |
| --- | --- | --- | --- | --- |
| BT1 | 20.35 | 17.14 | 20.89 | 0.82 |
| BT2 | 17.9 | 18.7 | 22.77 | 0.65 |
| BT3 | 24.97 | 18.53 | 19.1 | 0.05 |
| BT2-BT1 | -2.5 | -0.93 | -2.17 | 0.90 |
| BT3-BT1 | 3 | -0.83 | -2.7 | 0.10 |

Supplementary Table S4 Clinical characteristics of patients in the PR+SD and PD groups

| Characteristic | Total (n=160) | PR+SD (n=136) | PD (n=24) |
| --- | --- | --- | --- |
| Age |  |  |  |
| ＜65 | 87 (54.4) | 75 (55.1) | 12 (50.0) |
| ≥65 | 73 (45.6) | 61 (44.9) | 12 (50.0) |
| Sex |  |  |  |
| Male | 114 (71.2) | 95 (69.9) | 19 (79.2) |
| Female | 46 (28.8) | 41 (30.1) | 5 (20.8) |
| Smoking history |  |  |  |
| Never | 120 (75.0) | 105 (77.2) | 15 (62.5) |
| Current/former | 40 (25.0) | 31 (22.8) | 9 (37.5) |
| ECOG PS |  |  |  |
| 0 | 15 (9.4) | 12 (8.8) | 3 (12.5) |
| 1 | 135 (84.4) | 116 (85.3) | 19 (79.2) |
| 2 | 10 (6.2) | 8 (5.9) | 2 (8.3) |
| Histology |  |  |  |
| Adenocarcinoma | 125 (78.1) | 106 (77.9) | 19 (79.2) |
| Squamous cell carcinoma | 31 (19.4) | 26 (19.1) | 5 (20.8) |
| Other | 4 (2.5) | 4 (3.0) | 0 (0) |
| Clinical stage at diagnosis |  |  |  |
| IIIB-C | 21 (13.1) | 17 (12.5) | 4 (16.7) |
| IV | 139 (86.9) | 119 (87.5) | 20 (83.3) |
| Tumor size |  |  |  |
| ＜5cm | 99 (61.8) | 86 (63.2) | 13 (54.2) |
| ≥5cm | 46 (28.8) | 36 (26.5) | 10 (41.7) |
| Unable to measure | 15 (9.4) | 14 (10.3) | 1 (4.1) |
| NLR |  |  |  |
| ＜2.98 | 84 (52.5) | 72 (52.9) | 12 (50.0) |
| ≥2.98 | 76 (47.5) | 64 (47.1) | 12 (50.0) |

Supplementary Table S5 Comparison of cfDNA concentrations in patients in the PR+SD and PD groups

| Characteristic | Total | PR+SD | PD |
| --- | --- | --- | --- |
| BT1 cfDNA | 20.62  (13.15, 33.17) | 20.64  (13.03, 33.17) | 20.34  (14.10, 34.16) |
| BT2 cfDNA | 19.65  (11.79, 30.06) | 19.84  (11.83, 29.03) | 17.90  (11.71, 32.45) |
| BT3 cfDNA | 19.80  (11.76, 27.62) | 19.10  (10.86, 25.96) | 24.96  (16.36, 49.36) |
| BT2-BT1 cfDNA | -1.47  (-13.49, 7.68) | -1.06  (-13.11, 7.68) | -2.50  (-16.64, 6.44) |
| BT3-BT1 cfDNA | -1.41  (-11.81, 7.27) | -2.22  (-12.17, 6.27) | 3.01  (-7.41, 24.19) |
