# Supplementary figures and images for: Relationship between plasma circulating cell-free DNA concentration and treatment outcomes including prognosis in patients with advanced non-small cell lung cancer
Source: BMC Pulm Med. 2023 Sep 14;23:348. doi: 10.1186/s12890-023-02586-2 (PMC10503004; doi:10.1186/s12890-023-02586-2)

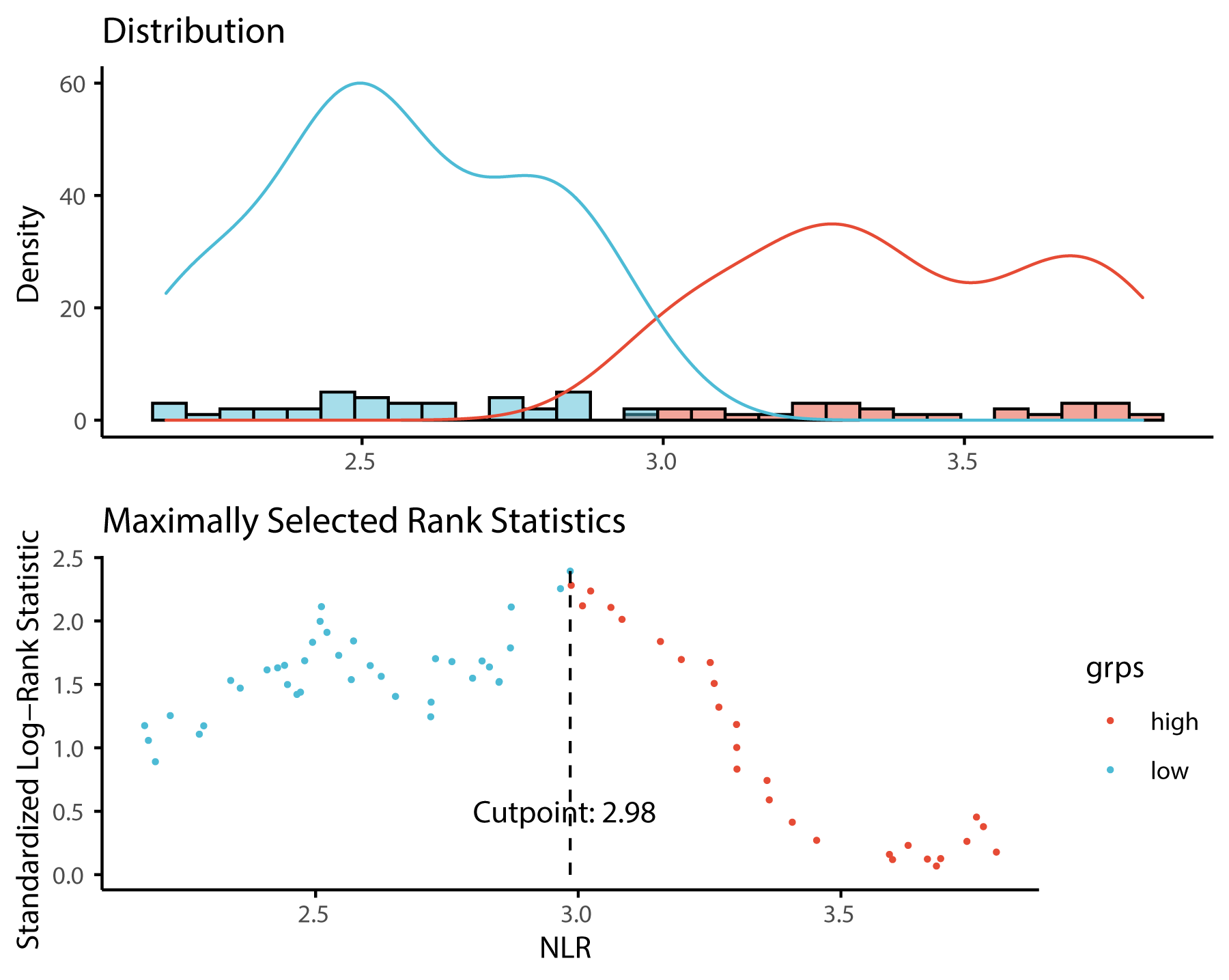

Supplement: Supplementary file 3 — Supplementary Material 3 [file 12890_2023_2586_MOESM3_ESM.png]

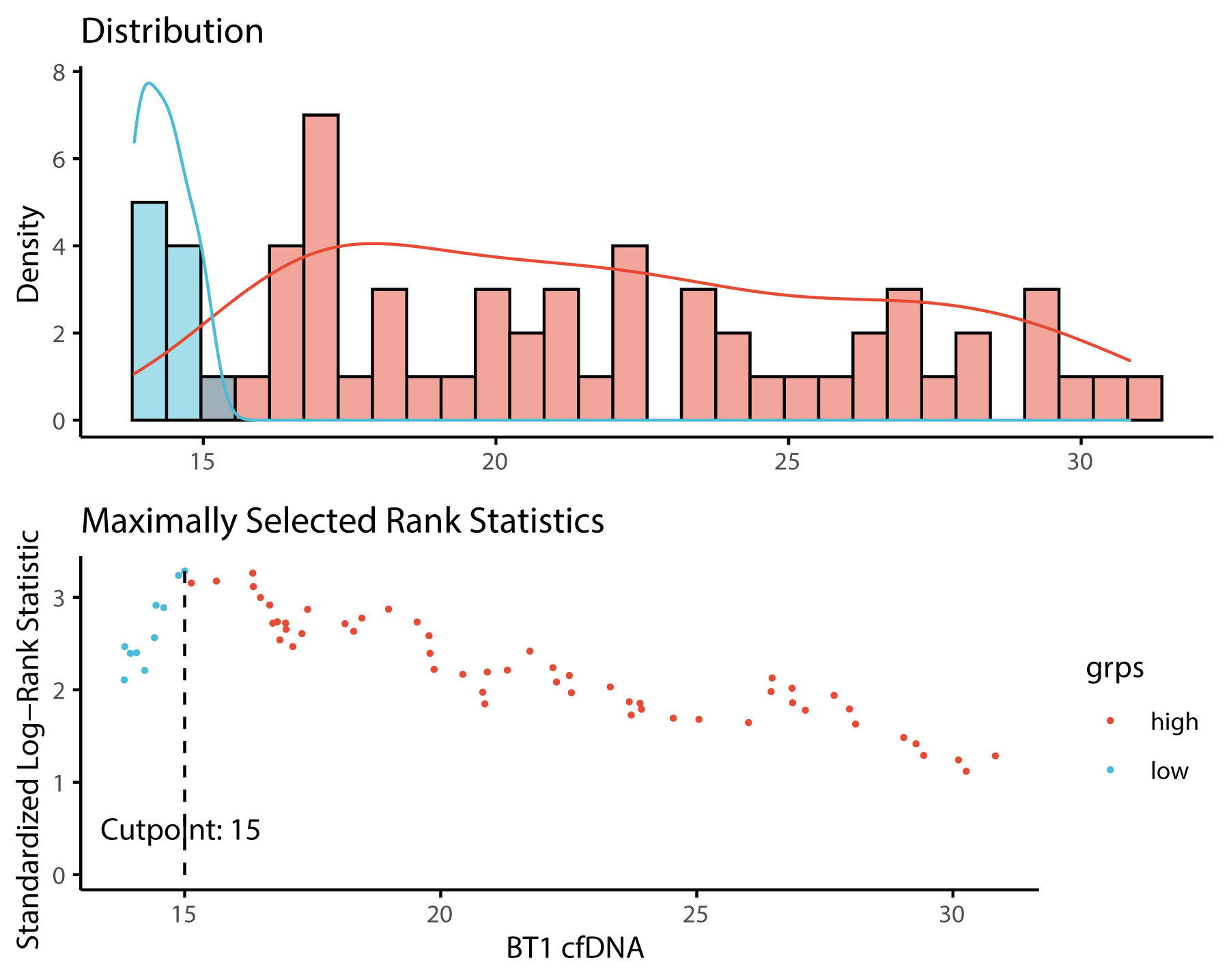

Supplement: Supplementary file 4 — Supplementary Material 4 [file 12890_2023_2586_MOESM4_ESM.png]

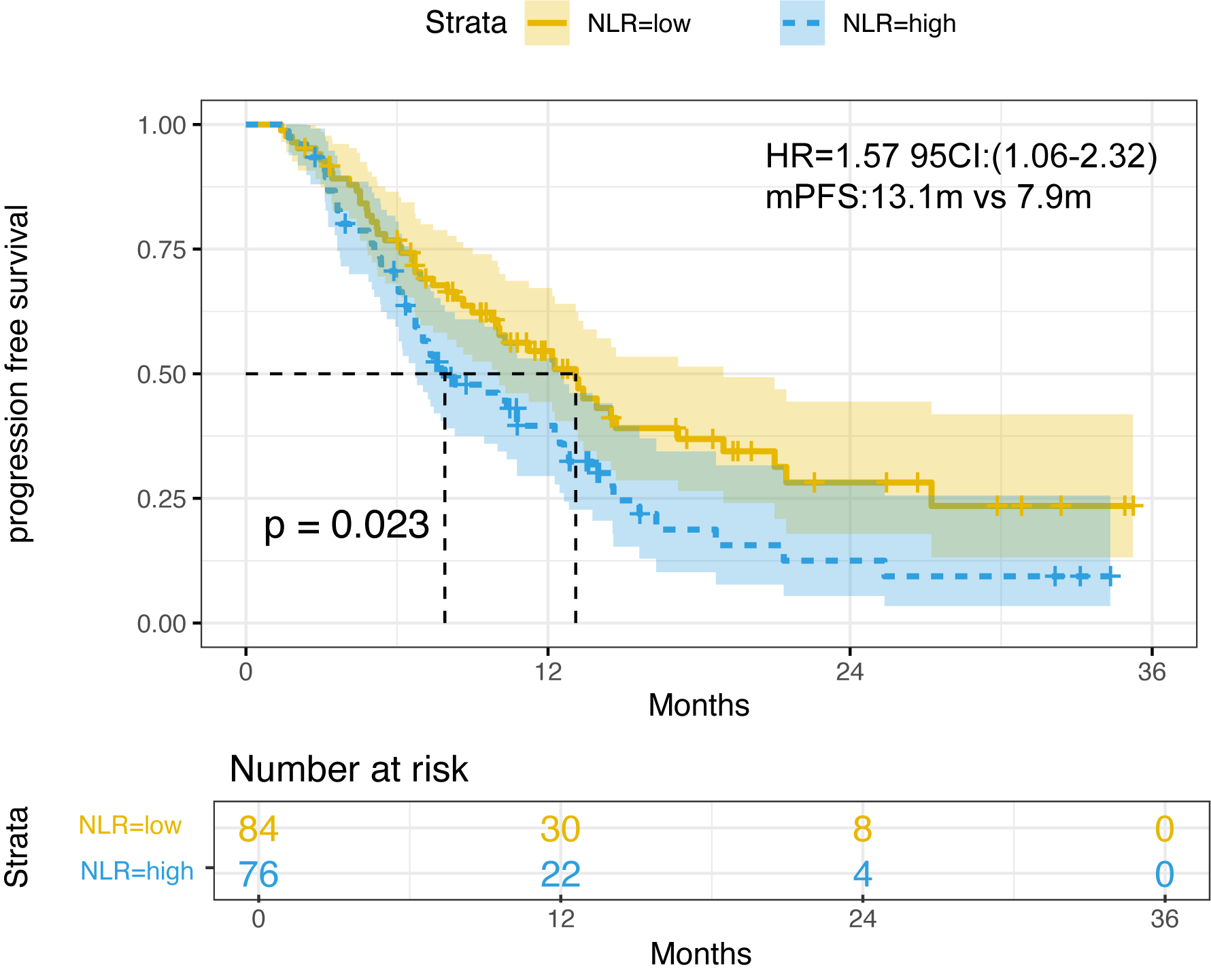

Supplement: Supplementary file 5 — Supplementary Material 5 [file 12890_2023_2586_MOESM5_ESM.png]

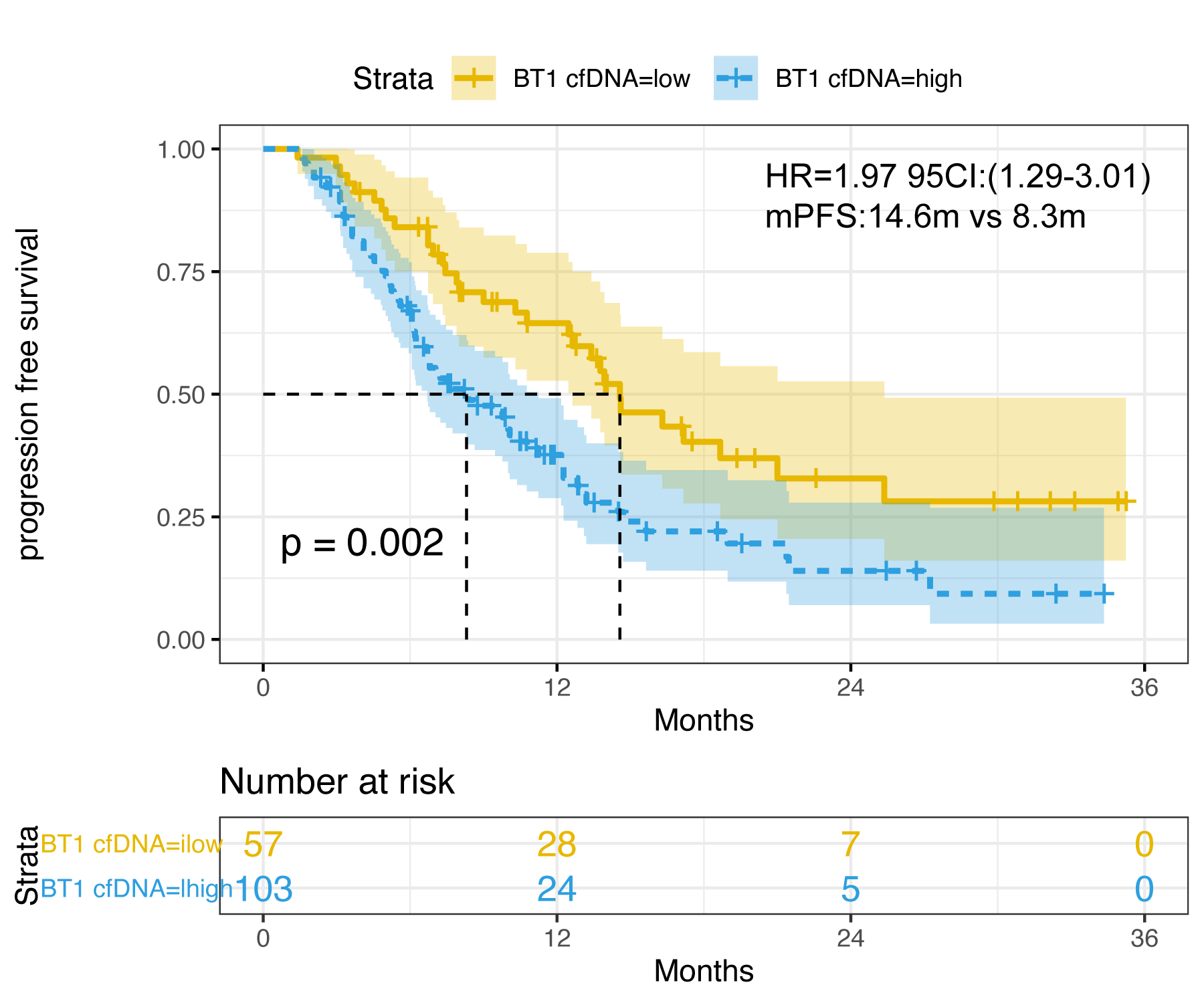

Supplement: Supplementary file 6 — Supplementary Material 6 [file 12890_2023_2586_MOESM6_ESM.png]

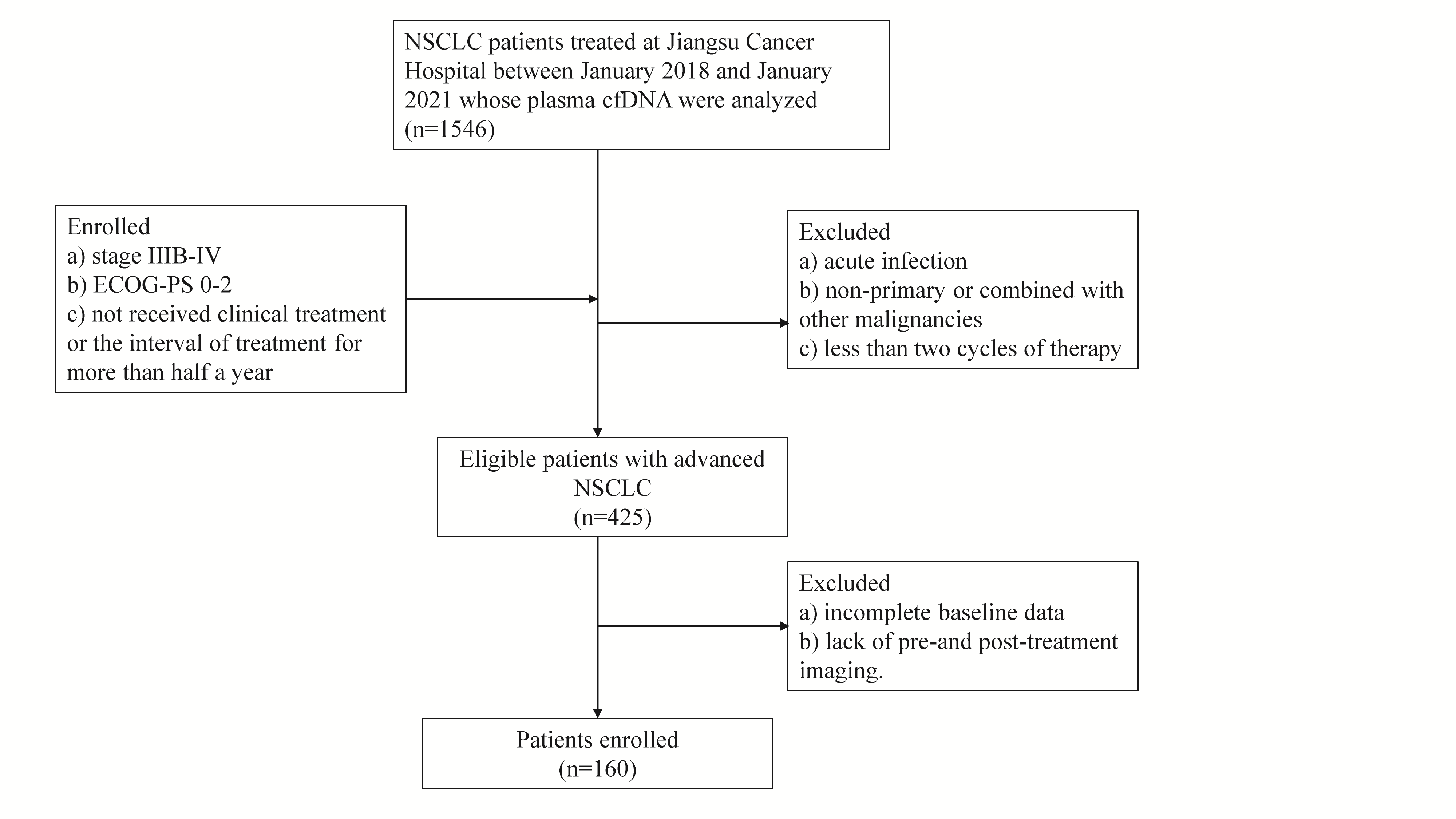

Supplement: Supplementary file 7 — Supplementary Material 7 [file 12890_2023_2586_MOESM7_ESM.png]

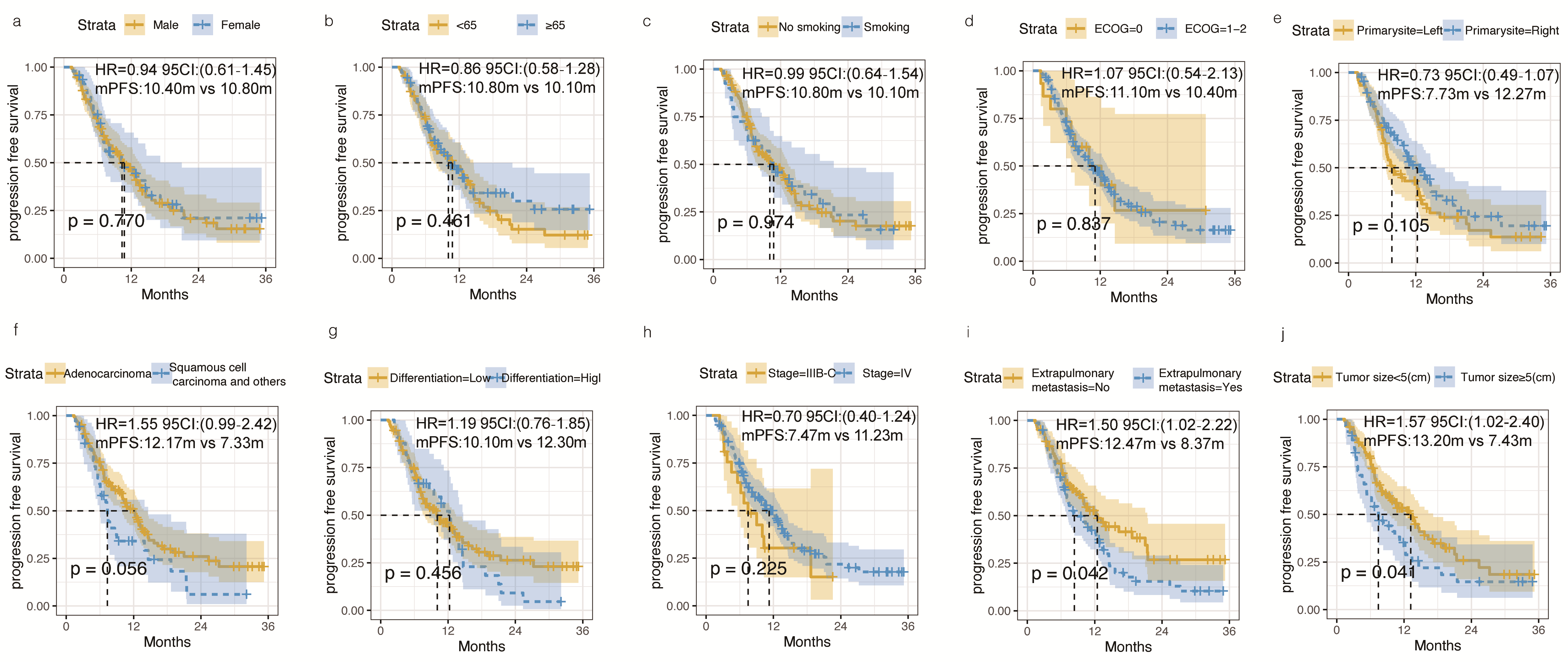

Supplement: Supplementary file 8 — Supplementary Material 8 [file 12890_2023_2586_MOESM8_ESM.png]

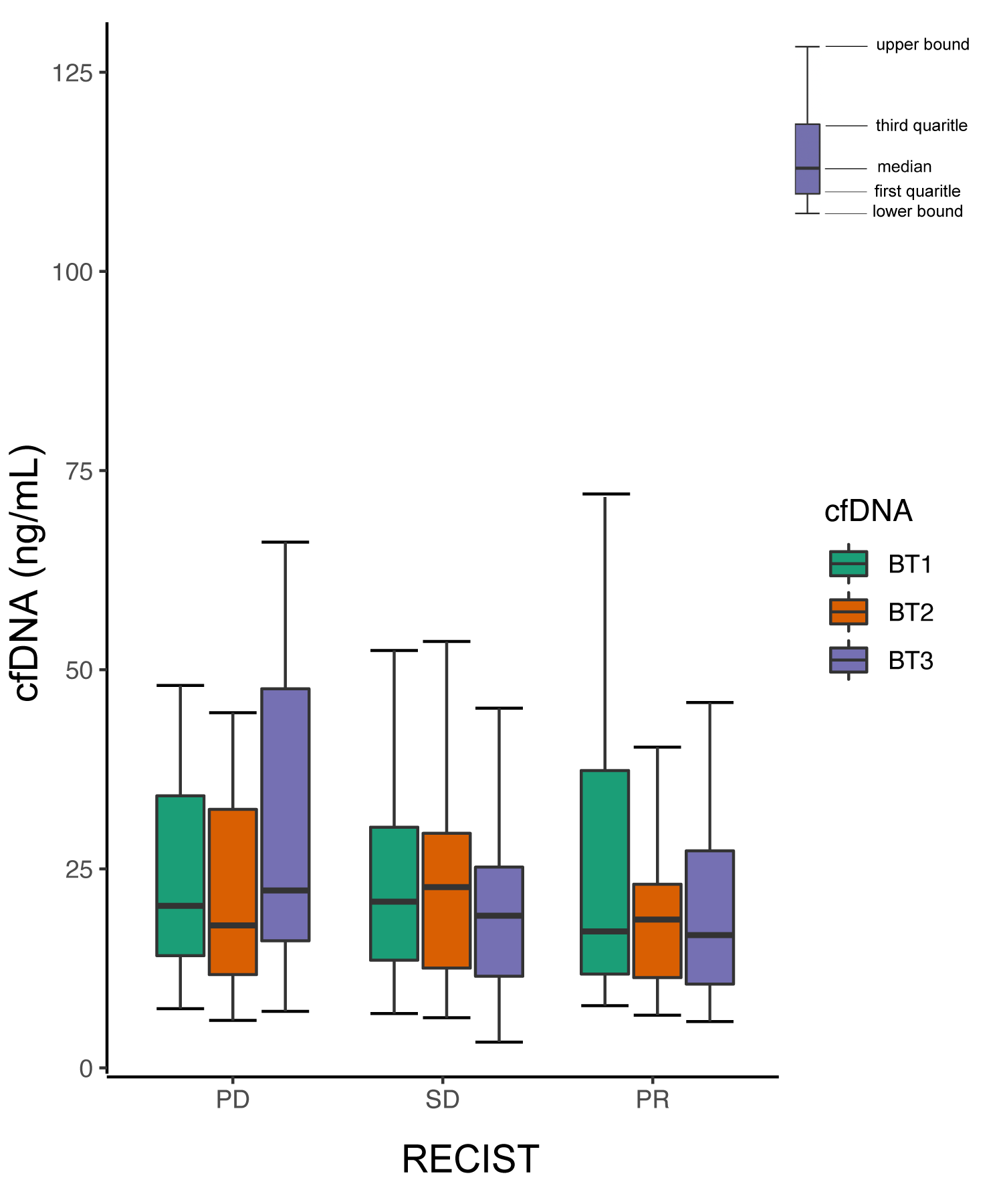

Supplement: Supplementary file 9 — Supplementary Material 9 [file 12890_2023_2586_MOESM9_ESM.png]
